# New Advance in Caspase-Independent Programmed Cell Death and its Potential in Cancer Therapy

**Published:** 2006-09

**Authors:** Rong Qi, Xin Yuan Liu

**Affiliations:** 1 *Institute of Biochemistry and Cell Biology, Shanghai Institutes for Biological Sciences, Chinese Academy of Sciences, Shanghai, China;*; 2 *College of Life Science & Technology, Shanghai Jiaotong University, Shanghai, China;*; 3 *Xinyuan Institute of Medicine and Biotechnology, Zhejiang Sci-Tech University, Hangzhou, China*

**Keywords:** apoptosis, autophagy, cancer therapy, caspase-independent programmed cell death

## Abstract

Caspase activation has been frequently viewed as synonymous with programmed cell death (PCcD); however, accumulating evidence showed that there existing caspase-independent PCcD pathways displaying morphologies that are not fully consistent with classical apoptosis. In this article, we will focus on the most recent progresses of different models of PCcD independent of caspases activity. Since some tumor cells can unexpectedly survive the activation of caspases, and tumor suppressor proteins that activate caspase-independent PCcD are commonly mutated in human cancer, the alternative cell death pathways are gaining increasing interest among cancer researchers. Though the mechanism of this cell death pathway is poorly understood, it is clear that a full understanding of the regulation of caspase-independent PCcD could provide new means of improving current diagnosis and promoting conceptual advances for the design of new therapeutic strategies for cancer therapy.

## INTRODUCTION

Programmed cell death (PCD) is a genetically regulated process of cell suicide that is crucial to the development, homeostasis and integrity of multicellular organisms (1). Acquired defects in signaling pathways leading to PCD are among the major hallmarks of cancer (2). Accordingly, cell death triggers that could induce PCD have gained increasing interest among cancer researchers. The best-defined cell death program is known as apoptosis (3), the most defining features of which are the activation of caspases, chromatin condensation and the display of phagocytosis markers on the cell surface, so that the majority of current anticancer therapies induce tumor cell death through the induction of apoptosis. However, accumulating evidence showed that there existing alternative PCD pathways without caspase activation (4-9). The heterogeneity of tumor cells with respect to their sensitivity to various death stimuli emphasizes the need for a better understanding of diverse modes of tumor cell death (10). Here the progress of caspase-independent PCD was reviewed and its potential in cancer therapy was discussed.

### The classification of PCD

Cell death is generally classified into two large categories: Programmed cell death (PCD) and necrosis. Different from necrosis (passive cell death without underlying regulatory mechanisms), PCD is cell death dependent on signals or activities within the dying cell (10). In 1972 Kerr and his colleagues identified a distinct subgroup of cell death characterized by nuclear and cytoplasmic shrink-age, which they termed ‘apoptosis’ (11). Since the term apoptosis was introduced on morphological grounds, this idea has been supported by ever increasing evidence and apoptosis has long been used as a synonym for PCD.

In fact, the existence of alternative types of PCD has ever been reported by Schweichel and Merker based on observations which they made in embryonic and fetal tissues of rats and mice in 1973 (12). But their classification was ignored until Clarke revisited and broadened it in 1990, resulting in the classification of physiological cell death into three morphological categories: “apoptotic” (Type I cell death), “autophagic” (Type II cell death), and “non-lysosomal vesiculate” (Type III cell death) (13). The three types of PCD have in common that they are executed by active cellular processes that can be intercepted by interfering with intracellular signaling. This distinguishes them from accidental necrosis. The salient features of the three main types of PCDs have been summarized in detail by Kitanaka (14). The type III cell death occurs through disintegration of cells into fragments without involvement of the lysosomal system and without marked condensation. Since researches based on this PCD type are seldom reported, this review will focus on the type I and II PCDs and the role of caspases in them.

### Type I cell death

Type I cell death is most likely identical to apoptosis, which is caspase-dependent and in some cases also caspase-independent. Classic apoptosis, as mentioned above, proceeds by cell shrinkage, chromatin condensation, nucleosomal DNA degradation and fragmentation of the cell into apoptotic bodies. Activation of the caspase family gives rise to these characteristic morphological features of apoptosis. During the 90s in 20th century, most investigators were convinced that caspases were responsible for cell death. Indeed, many cells die with apoptotic morphology and with documented activation of different caspases, but there are also many exceptions. In 1996, it was reported that Bax overexpression in Jurkat cells leads to an apoptotic-like death that is not blocked by caspase inhibitors. In these overexpressing Jurkat cells, caspase inhibitors do block DNA fragmentation and cleavage of substrates for caspases but these cells still die (5). Since then, more and more evidence identified the existence of caspase-independent PCD, some examples of which were shown in Table 1.

**Table 1:** PCD models not accompanied with caspase activation

Trigger	Cell type	Morphology	Protease

TNF-α (15)	human neutrophils	apoptotic-like and necrosis-like	calpain
BAY 43-9006 (16)	A2058 and SKMEL5 melanoma cells	apoptosis-like	Not known
LAPF (17)	L929 cells	apoptosis-like	cathepsin D
Histone deacetylase inhibitors (18)	IMIM-PC-1, IMIM-PC-2 and RWP-1	apoptosis-like	Omi/HtrA2
Imatinib mesylate (19)	human leukemic cells	necrosis-like	Omi/HtrA2
CI-1033 (20)	DiFi cells	apoptosis like	cathepsin D

Several other proteases have been described to be able to execute PCD, including calpains, cathepsins B, D, L, Omi/HtrA2 and granzymes A, B etc. These proteases often cooperate with caspases in classic apoptosis, but they can also trigger PCD and bring about many of the morphological changes characteristic of apoptosis in a caspase-independent manner (3, 10, 21). Moreover, the various forms of caspase-independent cell death cannot readily be classified as “apoptosis” or “necrosis”, and alternative types of PCD according to the degree of chromatin condensation have been described (3). Apoptosis-like PCD is PCD characterized by chromatin condensation and display of phagocytosis recognition molecules prior to the lysis of the plasma membrane. Chromatin condenses to lumpy masses that are less compact than in apoptosis (stage 1 chromatin condensation). Any degree and combination of other apoptotic features can be found. Apoptosis-like PCD can be dependent or independent of caspases. Necrosis-like PCD is PCD in the absence of chromatin condensation or, at best, with chromatin clustering to loose speckles. A varying degree of apoptosis-like changes including phosphatidylserine exposure might occur prior to the lysis. Aborted apoptosis and type II cell death usually fall into this category (10).

However, caspase-dependent and –independent PCD aren’t exclusive. Cells can always respond to both of them under different stimulations. NF-κB inactivation and inhibition of RNA synthesis can both sensitize RALA255-10G hepatocytes to TNF-α toxicity through apoptosis-like PCD, although ActD-induced (inhibition of RNA synthesis) hepatocyte sensitization to TNF-α cytotoxicity occurs through a caspase-independent pathway while Ad5IκB, that blocks NF-κB activation induce cell death through caspase-dependent pathaway (22). It is common that a cell death trigger can induce caspase-dependent PCD, while in the absence of caspases, owing to the pan-caspase inhibitor, gene mutation, or something else, caspase-independent PCD will take the dominant role (5, 19, 23). AD5–10(anti-human DR5 monoclonal antibody) activates the conventional caspase cascade in Jurkat cells and causes a classical caspase-dependent cell death. The activation of the caspase cascade is the basic function of DR5. Interestingly, Z-VAD (pan-caspase inhibitor) failed to inhibit cell death induced by AD5–10, indicating that AD5–10 activated a caspase-independent signal pathway in addition to the classical caspase-dependent pathway (23). The process of caspase-independent cell death is much slower than the process of caspase-dependent cell death, therefore, only when the caspase-cascade was inhibited can we see the caspase-independent cell death induced by AD5-10 (23). It was implied that cells are in trouble can activate numerous PCD toward selfdestruction. Intrinsic or extrinsic routes to effector caspase activation are frequently the most rapid and efficient. If neither of these routes is immediately available, owing to mutation, genetic manipulation, inhibitor, or the biology of the cell, other routes may be followed, leading to variant forms of cell death that may display one or more characteristics of apoptosis (9). Therefore, different types of cell death may develop concomitantly to accomplish the important tasks of tissue remodeling and other developmental processes that require genetically controlled.

### Type II cell death

Type II cell death or autophagic cell death is characterized by the appearance of double- or multiple- membrane cytoplasmic vesicles engulfing bulk cytoplasm and/or cytoplasmic organelles such as mitochondria and endoplasmic reticulum. Autophagic vesicles and their contents are destroyed by the lysosomal system of the same cell. As a consequence, the cell ‘cannibalizes itself’ from inside (autophagia is self-digestion in Greek).

Unlike type I PCD, caspases are not activated in this type of cell death, the nucleus stays intact until the late phases of cell death and cellular fragmentation is not observed (24). Thus, the occurrence of caspase-independent cell death concomitant with an increased autophagic activity may be indicative of autophagic type II cell death. Several morphological characteristics detecting autophagic activity have been indicated. The demonstration of the autophagic vesicles by electron microscopy is still the ‘golden standard’ for assessing the autophagic activity. Increased autophagic/lysosomal activity may also be demonstrated by MDC staining or by the measurement of the degradation rate of radiolabeled long-lived proteins. The change in the intracellular localization of LC3 protein and its increased electrophoretic mobility upon LC3 recruitment to autophagic membranes provide the first molecular marker-based methods for the detection of autophagic activity (24). But it needs more precise definition. Like apoptotic programmed cell death, autophagy is a highly conserved process and an essential part of growth regulation and maintenance of homeostasis in multicellular organisms. Autophagy-related genes were first identified in yeast, but homologs are found in all eukaryotes. Based on the ways substrates reach the lysosomal lumen, three major forms of autophagy have been described in mammalian cells: macroautophagy, microautophagy and chaperone-mediated autophagy (25), while the exact mechanism underlying autophagy is poorly understood until now. Recent studies have identified many components that are required to drive this complicated cellular process. Mediators of class I and class III PI3 kinase signaling pathways and trimeric G proteins play major roles in regulating autophagosome formation during the stress response (26).

Thus, PCD may be further divided into two subgroups according to the involvement of caspases in cell death regulation: caspase-dependent and caspase-independent PCD. As we have discussed before, Type I cell death comprises both of them. Caspase activity is indispensable to the most classic form-apoptosis. But in its absence or failure, there are many other default pathways for cell self-destruction. Type II cell death or autophagic cell death is another caspase-independent PCD type (24). The type I and type II PCD should not be considered as mutually exclusive phenomena. Rather, they appear to reflect a high degree of flexibility in a cell’s response to changes of environmental conditions, both physiological and pathological (27). We summarized the different types of PCD, their dependence on caspases and different morphology in Table 2.

**Table 2:** Classification of PCD, their dependence on caspases and different morphology proposed in this review

Programmed Cell Death (PCD)			
Classification	Type I cell death (apoptotic)	Type II cell death (autophagic)	Type III cell death (non-lysosomal vesiculate)

Dependence on caspases	caspase-dependent and in some cases also caspase-independent	caspase-independent	Not known
Morphology	Apoptosis-like or Necrosis-like	Necrosis-like	Necrosis-like

Nevertheless, these present study might be just the tip of the iceberg for the complexity of this alternative death signaling pathway. In fact, except autophagy, which has been identified with relatively explicit morphological characteristics and molecular mechanism, almost all evidence concerning caspase-independent death is defined in indirect terms. The term ‘caspase-independent PCD’ just means these cell deaths can’t be rescued by caspases inactivation. In fact, the mechanismes underlying them share little in common. At present, it is generally accepted that analogous to classic apoptosis, MMP controlled by Bcl-2 family proteins resides at the heart of several alternative death pathways (10). And besides the several proteases mentioned above, a few molecules may play a central role in caspase-independent PCD, for instance: AIF(Apoptosis-inducing Factor) (28-30), Endonuclease G etc (31).

### Caspase-independent PCD for cancer therapy

Many studies have demonstrated the importance of caspase-independent cell death pathways in injury, degenerative diseases and tumor tissue. The discovery and understanding of caspase-independent PCD will open new perspectives for the treatment of cancer.

First of all, the major challenge in treating cancer is that many tumor cells carry mutations in key apoptotic genes such as p53, Bcl family proteins or those affecting caspase signaling. With respect to the unexpected ability of some cells to survive the activation of pro-apoptotic caspases activation, it would be dangerous for the organism to depend on a single protease family for the clearance of unwanted and potentially harmful cells (3). Caspase-independent PCD, which can be triggered by many factors, provides new options to kill tumor cells. Experimental gene-therapy approaches also point to it as promising targets for tumor therapy. For example, Caspase-independent apoptosis-like PCD could be activated by diazepam-induced mitotic failure in HeLa cells, but not in human primary fibroblasts (7). Deregulated apoptosis in cancer cells will likely not oppose effective CRAd-induced cell death. Their mechanism of tumor cell killing is therefore different from conventional therapies in which apoptosis activation contributes to cell death and may provide an explanation for the potency of CRAds in killing chemo-resistant tumor cells (32). The depletion of Hsp70 by adenovirus-mediated transfer of antisense cDNA induces caspase-independent death of tumorigenic cells while non-tumorigenic cells are unaffected, suggesting that Hsp70 has cancer-specific function(s) (33). vitamin D analogues, which could induce caspase-independent PCD in breast cancer cells, already has advanced into phase III clinical trials (34). Our group also found that ZD55-XAF1, a newly identified tumor suppressor gene carried by a conditionally replicative adenovirus could potently and specifically kill tumor cells *in vitro* and *in vivo* via Caspase-independent PCD (data not published).

Secondly, emerging evidence suggests that caspase-independent PCD is also deregulated in cancer. For example, Hsp70, which appears as a potent survival protein capable of protecting cells from a wide variety of lethal stimuli, is commonly overexpressed in solid tumors (35). Therefore downregulated expression of Hsp70 to induce caspase-independent PCD would be a preferable choice for these tumors. Bin1, a tumour-suppressor protein that is often missing or functionally inactivated in human cancer, can activate a caspase-independent apoptosis-like death process that is blocked by a serine protease inhibitor or simian virus large T antigen, but not by overexpression of Bcl-2 or inactivation of p53 (36).

Malignant transformation is also frequently associated with suppression of autophagy (24, 27). The relationship between autophagic activity and cancer was firstly indicated in this respect that the autophagic capacity of cancer cell lines was often lower than their normal counterparts and failed to be increased in response to autophagy triggers (38). However, advanced research found that autophagy probably factors into both the promotion and prevention of cancer, and its role may be altered during tumor progression. At the early stage of tumour development, autophagy functions as a tumour suppressor. Expression of beclin 1, a mammalian orthologue of the yeast autophagy-related gene Atg6, reduces tumorigenic capacity through induction of autophagy (11). Endogenous Beclin 1 protein expression is frequently low in human breast epithelial carcinoma cell lines and tissue, but is expressed ubiquitously at high levels in normal breast epithelia. Stable transfection of Beclin 1 in MCF7 breast cancer cells reduced the malignant phenotype, slowed down the proliferation rate and reduced their growth in soft agar. Furthermore, the tumorigenic capacity in nude mice of MCF-7 overexpressing the Beclin 1 gene was severely attenuated (39). On the contrary, mice that are Beclin 1+/- display a remarkable increase in the incidence of lung cancer, hepatocellular carcinoma and lymphoma (37, 40). At advanced stages of tumor development, autophagy promotes tumor progression. The tumor cells that are located in the central area of the tumor mass undergo autophagy to survive low-oxygen and low-nutrient conditions (38). So autophagy is a double-edged sword in cancer therapy. When tumor cells induce protective autophagy, it needs to be inhibited to sensitize tumor cells to the treatment by activating apoptosis. On the other hand, induction of autophagic cell death can also have a therapeutic value (38). Besides what discussed above, autophagic type II cell death may be a means to inhibit angiogenesis (41). The most direct evidence is reported by Chau, YP *et al*. in 2003. The angiogenesis inhibitor endostatin induced endothelial cell death, which showed characteristics of autophagic type II cell death, including caspase-independence, increased autophagic activity and sensitivity to 3-MA. Most importantly, for the heterogeneity of cancer, combination of caspase-dependent and -independent therapies provides a more efficient approach to block the commonly observed therapy resistance of transformed cells. The human pancreatic cell line L3.6 is more effectively killed following treatment with combination of two chemotherapeutic agents, paclitaxel and daunorubicin, which could cause death through at least two pathways, a caspase-dependent and caspase-independent cell death (42).

## CONCLUSIONS

With the emergency of ‘caspase-independent PCD’, it is more clear that death signaling in tumor cells appears much more complex than originally suggested by the simple caspase activation model. Cells that are under pressure to die have many options and routes to death. If a route involving caspases is blocked, the cell can activate other mechanisms with multiple morphologies to accomplish its imperative. A better understanding of diverse modes of tumor cell death will help to avoid ineffective treatment and provide a molecular basis for the new strategies targeting caspase-independent death pathways in cancer therapy.
